# Novel signal peptides improve the secretion of recombinant *Staphylococcus aureus* Alpha toxin_H35L_ in *Escherichia coli*

**DOI:** 10.1186/s13568-017-0394-1

**Published:** 2017-05-12

**Authors:** SooJin Han, Shushil Machhi, Mark Berge, Guoling Xi, Thomas Linke, Ronald Schoner

**Affiliations:** 1 Cell Culture and Fermentation Sciences, MedImmune Inc., Gaithersburg, MD 20878 USA; 2 Purification Process Sciences, MedImmune Inc., Gaithersburg, MD 20878 USA; 3 BioProcess Sciences, Qilu Puget Sound Biologics, Bothell, WA 98021 USA

**Keywords:** Alpha toxin, *Escherichia coli*, Extracellular secretion, Periplasmic translocation, Signal peptides, Signal peptide recognition particle, *Staphylococcus aureus*, The SEC pathway, The SRP pathway

## Abstract

Secretion of heterologous proteins into *Escherichia coli* cell culture medium offers significant advantages for downstream processing over production as inclusion bodies; including cost and time savings, and reduction of endotoxin. Signal peptides play an important role in targeting proteins for translocation across the cytoplasmic membrane to the periplasmic space and release into culture medium during the secretion process. Alpha toxin_H35L_ (AT_H35L_) was selected as an antigen for vaccine development against *Staphylococcus aureus* infections. It was successfully secreted into culture medium of *E. coli* by using bacterial signal peptides linked to the N-terminus of the protein. In order to improve the level of secreted AT_H35L_, we designed a series of novel signal peptides by swapping individual domains of modifying dsbA and pelB signal peptides and tested them in a fed-batch fermentation process. The data showed that some of the modified signal peptides improved the secretion efficiency of AT_H35L_ compared with *E. coli* signal peptides from dsbA, pelB and phoA proteins. Indeed, one of the novel signal peptides improved the yield of secreted AT_H35L_ by 3.5-fold in a fed-batch fermentation process and at the same time maintained processing at the expected site for signal peptide cleavage. Potentially, these new novel signal peptides can be used to improve the secretion efficiency of other heterologous proteins in *E. coli*. Furthermore, analysis of the synthetic signal peptide amino acid sequences provides some insight into the sequence features within the signal peptide that influence secretion efficiency.

**Electronic supplementary material:**

The online version of this article (doi:10.1186/s13568-017-0394-1) contains supplementary material, which is available to authorized users.

## Introduction

*Escherichia coli* offers many advantages as a production organism, including growth on inexpensive carbon sources, rapid biomass accumulation, amenability to high cell-density fermentations and simple process scale-up (Mergulhao et al. 2005). Recombinant proteins can be produced in *E. coli* as intracellular inclusion bodies; however secretion into the extracellular environment is preferred as it simplifies downstream purification processes, protects recombinant proteins from proteolysis by cytoplasmic or periplasmic proteases, reduces endotoxin levels and contamination of the product by host proteins, and enhances biological activity (Gottesman 1996). In *E. coli*, proteins normally do not secreted into the extracellular circumstance except for a few classes of proteins such as toxin and hemolysin. However, small proteins are frequently released into the culture medium depends on the characteristics of signal sequences and proteins (Choi and Lee 2004; Tong et al. 2000). Many studies have therefore been carried out to improve the secretion efficiency of recombinant proteins in *E. coli* expression systems (Baneyx and Mujacic 2004; Cornelis 2000; Klatt and Konthur 2012; Mergulhao et al. 2005).

In *E. coli*, the Sec-dependent (Sec) secretion pathway is the general secretion route and signal peptides linked to the N-terminus of recombinant proteins play a critical role in translocation and secretion. Unfolded precursor proteins are translocated across the cytoplasmic membrane with concomitant cleavage of the signal peptide by signal peptidase and released into the periplasmic space where they fold into their native structure (Green and Mecsas 2016; Mergulhao et al. 2005; Valent et al. 1998). The further export of recombinant proteins into the extracellular environment has been reported for a number of proteins (Kotzsch et al. 2011; Qian et al. 2008). The Sec secretion pathway can also utilize a co-translational mechanism of export that couples translation of proteins by the ribosome with secretion through the SecYEG channel [the signal peptide recognition particle (SRP) pathway]. The SRP pathway relies on the SRP particle, which recognizes an N-terminal signal peptide with highly hydrophobic core during protein secretion and the binding affinity of the SRP particle for signal peptides increases with the hydrophobicity of the h-region of signal peptides (Green and Mecsas 2016; Nilsson et al. 2015).

Signal peptides are short peptides (generally 20–30 amino acid residues in length) and have three distinguishable structural domains with different functions; an amino-terminal region with a net positive charge (the n-region) followed by a hydrophobic region (the h-region) and a protease recognition sequence (the c-region) with a preference for small residues at the −3 (P3) and −1 (P1) positions relative to the cleavage site (Fekkes and Driessen 1999; Paetzel et al. 2000; Paetzel 2014). In order to improve secretion of recombinant proteins in *E. coli* expression systems, a number of heterologous signal peptides have been evaluated (Velaithan et al. 2014; Jonet et al. 2012; Ismail et al. 2011; Low et al. 2011; Nagano and Masuda 2014). These studies demonstrated that the hydrophobic region in the signal peptide plays an important role for protein translocation across the bacterial cytoplasmic membrane due to the interaction of the h-region with the membrane during protein translocation. Several studies have also reported that translocation efficiency increases with the length and hydrophobicity of the h-region, and a minimum hydrophobicity is required for their secretion function (Duffy et al. 2010; Ryan et al. 1993; Wang et al. 2000).

The pore-forming α-hemolysin protein, also known as α-toxin (AT), is produced by the majority of *Staphylococcus aureus* (*S. aureus*) serotypes and secreted as a water soluble monomer (Craven et al. 2009; Kennedy et al. 2010; Ragle and Bubeck 2009; Wardenburg and Schneewind 2008). The AT polypeptide is processed through the secretory machinery to yield a mature ~33 kDa protein of 293 amino acids (Berube and Wardenburg 2013). It is one of the most well-characterized bacterial virulence factors. AT oligomerizes into a heptameric structure on the host cell membrane creating a pore structure resulting in cell lysis. However, substitution of histidine 35 with leucine (AT_H35L_) leads to the loss of hemolytic activity of AT (Menzies and Kernodle 1994, 1996; O'Reilly et al. 1986; Ragle and Bubeck 2009). Since there are no vaccines available for the prevention of *S. aureus* infections, the mutant AT_H35L_ has been investigated as a vaccine target (Menzies and Kernodle 1996; Ragle and Bubeck 2009) and, a previous inflammation study has used mutant AT_H35L_ isolated from crude *E. coli* (Craven et al. 2009; O'Reilly et al. 1986; Saito et al. 2009). In this study, we designed novel signal peptides that significantly improved the secretion of the AT_H35L_ protein into culture medium in *E. coli* and maintained proper cleavage processing to give the mature AT_H35L_ protein sequence. We also demonstrated that the position of amino acid residues in the h-region is a potentially important factor affecting secretion of recombinant proteins.

## Materials and methods

### *Escherichia coli* strains and growth conditions

*Escherichia coli* strains BL21 (DE3) [fhuA2 [lon] ompT gal (λ sBamHIo ∆*Eco*RI-B int: *lac*I: PlacUV5::T7 gene1) i21 ∆nin5)[dcm]∆hsdS] and BL21 Star™ (DE3) [F^−^ *omp*T *hsd*S_B_ (r_B_^−^, m_B_^−^) *galdcmrne*131 (DE3)] were chosen as hosts for recombinant protein expression. Recombinant cells were cultured in seed medium (20 g/L yeast extract) or rich growth medium (20.3 g/L yeast extract (BioSpringer, Milwaukee, WI, USA) 10.1 g/L sodium sulfate anhydrous (JT baker, Center Valley, PA, USA) and 7 g/L K_2_HPO_4_ (JT baker, Center Valley, PA, USA) both supplemented with 50 μg/mL kanamycin (Sigma, St. Louis, MO, USA) for expression of recombinant AT_H35L_ proteins at 30 °C.

### Construction of expression plasmids

Expression vector, pJ411, provided by DNA 2.0 Inc. (Menlo Park, CA, USA) was used for T7 promoter driven-expression of signal peptide variants linked to the AT_H35L_ gene. The kanamycin resistance gene was used as a selection marker. To generate AT_H35L_ gene (Gene bank accession no. KY474302), wild-type of AT gene (Gene bank accession no. CP006838.1) was mutated by substitution of histidine 35 with leucine and the mutant was codon-optimized for expression in *E. coli.* Nucleic acid and amino acid sequences of the codon-optimized AT_H35L_ gene used in this study are shown in Table 1. Codon-optimization of nucleic acid sequences for expression in *E. coli*, gene synthesis and DNA sequencing analysis of AT_H35L_ with different signal peptides were performed by DNA 2.0 Inc (Menlo Park, CA, USA). A summary of all signal peptides and recombinant plasmids used in this study is shown in Table 2 and nucleic acid sequences of all signal peptides used in this study are summarized in Table 3.

**Table 1:** Nucleic acid and amino acid sequences of AT_H35L_ used in this study (Gene bank accession no. KY474302)

**1**	GCAGACAGCGACATC AACATTAAGACTGGT ACCACCGACATCGGC AGCAATACGACCGTT AAAACCGGCGACCTG
	A D S D I N I K T G T T D I G S N T T V K T G D L
**76**	GTGACCTACGATAAA GAGAATGGCATGTTG AAAAAAGTTTTCTAC TCTTTTATCGATGAT AAGAATCACAACAAA
	V T Y D K E N G M L K K V F Y S F I D D K N H N K
**151**	AAGCTGCTGGTCATT CGTACGAAGGGCACC ATCGCGGGTCAGTAT CGCGTCTACTCCGAA GAGGGCGCGAACAAG
	K L L V I R T K G T I A G Q Y R V Y S E E G A N K
**226**	AGCGGTCTGGCTTGG CCGAGCGCATTTAAG GTCCAGCTGCAACTG CCTGATAACGAAGTT GCGCAGATTAGCGAC
	S G L A W P S A F K V Q L Q L P D N E V A Q I S D
**301**	TACTACCCACGCAAT AGCATTGACACCAAA GAGTATATGAGCACC CTGACGTATGGCTTC AATGGTAACGTGACC
	Y Y P R N S I D T K E Y M S T L T Y G F N G N V T
**376**	GGCGACGACACGGGT AAGATCGGTGGTCTG ATCGGCGCCAATGTG AGCATCGGTCATACG CTGAAATATGTTCAG
	G D D T G K I G G L I G A N V S I G H T L K Y V Q
**451**	CCGGACTTCAAGACG ATTTTGGAGTCCCCG ACGGACAAAAAAGTT GGCTGGAAAGTGATT TTCAACAACATGGTC
	P D F K T I L E S P T D K K V G W K V I F N N M V
**526**	AATCAAAATTGGGGT CCGTACGATCGTGAC AGCTGGAACCCGGTG TATGGTAATCAACTG TTTATGAAAACCCGC
	N Q N W G P Y D R D S W N P V Y G N Q L F M K T R
**601**	AACGGTTCTATGAAA GCGGCCGACAACTTC CTGGATCCGAATAAG GCTAGCTCCCTGCTG TCGAGCGGTTTTAGC
	N G S M K A A D N F L D P N K A S S L L S S G F S
**676**	CCGGATTTTGCAACG GTGATTACCATGGAC CGTAAGGCGAGCAAG CAACAGACCAATATC GACGTCATTTACGAA
	P D F A T V I T M D R K A S K Q Q T N I D V I Y E
**751**	CGTGTTCGTGATGAT TATCAGCTGCACTGG ACTAGCACCAACTGG AAGGGTACCAACACC AAGGATAAATGGATT
	R V R D D Y Q L H W T S T N W K G T N T K D K W I
**826**	GATCGCTCAAGCGAA CGTTACAAGATCGAT TGGGAGAAAGAAGAG ATGACGAACTAA
	D R S S E R Y K I D W E K E E M T N *

**Table 2:** Amino acid sequences of bacterial and novel signal peptides used in this study

Plasmid ID	Signal peptide ID	The n-region^a^	The h-region^b^	The c-region^c^
DsbAss_AT_H35L_	DsbAss	MKKI (+2)	WLALAGLVL	AFSASA
PelBss_AT_H35L_	PelBss	MKYLLP (+1)	TAAAGLLLLA	AQPAMA
PhoAss_AT_H35L_	PhoAss	MKQST (+1)	IALALLPLL	FTPVTKA
NTss_AT_H35L_	NTss^d^	MKTH (+1.1)	IVSSVTTTLLLGSILMN	PVANA
149153	NSP1	MKYLLP (+1)	WLALAGLVL	AFSASA
149154	NSP2	MKKI (+2)	TAAAGLLLLA	AFSASA
149155	NSP3	MKKI (+2)	W^1^L^2^A^3^L^4^A^5^G^6^L^7^V^8^L^9^	AQPAMA
182988	NSP3a	MKKI (+2)	L^9^V^8^L^7^G^6^A^5^L^4^A^3^L^2^W^1^	AQPAMA
182989	NSP3b	MKKI (+2)	W^1^L^2^A^3^L^7^V^8^L^9^L^4^A^5^G^6^	AQPAMA
182990	NSP3c	MKKI (+2)	L^4^A^5^G^6^W^1^L^2^A^3^L^7^V^8^L^9^	AQPAMA
182991	NSP3d	MKKI (+2)	L^7^V^8^L^9^L^4^A^5^G^6^W^1^L^2^A^3^	AQPAMA
149156	NSP4	MKKI (+2)	T^1^A^2^A^3^A^4^G^5^L^6^L^7^L^8^L^9^A^10^	AQPAMA
187441	NSP4a	MKKI (+2)	L^6^L^7^L^8^L^9^G^5^T^1^A^2^A^3^A^4^A^10^	AQPAMA
187442	NSP4b	MKKI (+2)	LLLLLLLLLL	AQPAMA
187443	NSP4c	MKKI (+2)	AAAAAAAAAA	AQPAMA
149157	NSP5	MKYLLP (+1)	WLALAGLVL	AQPAMA
149158	NSP6	MKYLLP (+1)	TAAAGLLLLA	AFSASA

*ss* stands for signal sequence

^a^Numbers in parentheses indicate the positive net charge in the n-region

^b^Numbers indicate the position of amino acids in the h-region

^c^The cleavage sites are underlined in the c-region

^d^AT native signal sequence

**Table 3:** Nucleic acid sequences of bacterial and novel signal peptides used in this study

Signal peptide	Nucleic acid sequences (5′ to 3′)
DsbAss	ATGAAAAAGATTTGGCTGGCGCTGGCTGGTTTAGTTTTAGCGTTTAGCGCATCGGCG
PelBss	ATGAAATACCTGCTGCCGACCGCTGCTGCTGGTCTGCTGCTCCTCGCTGCCCAGCCGGCGATGGCG
PhoAss	ATGAAACAAAGCACTATTGCACTGGCACTCTTACCGTTACTGTTTACCCCTGTGACAAAAGCG
NTss	ATGAAAACACATATAGTCAGCTCAGTAACAACAACACTATTGCTAGGTTCCATATTAATGAATCCTGTCGCTAATGCC
NSP1	ATGAAATACCTGCTGCCGTGGCTGGCGCTGGCTGGTTTAGTTTTAGCGTTTAGCGCATCGGCG
NSP2	ATGAAAAAGATTACCGCTGCTGCTGGTCTGCTGCTCCTCGCTGCGTTTAGCGCATCGGCG
NSP3	ATGAAAAAGATTTGGCTGGCGCTGGCTGGTTTAGTTTTAGCCCAGCCGGCGATGGCG
NSP3a	ATGAAAAAGATTTTAGTTTTAGGTGCTCTGGCGCTGTGGGCCCAGCCGGCGATGGCG
NSP3b	ATGAAAAAGATTTGGCTGGCGTTAGTTTTACTGGCTGGTGCCCAGCCGGCGATGGCG
NSP3c	ATGAAAAAGATTCTGGCTGGTTGGCTGGCGTTAGTTTTAGCCCAGCCGGCGATGGCG
NSP3d	ATGAAAAAGATTTTAGTTTTACTGGCTGGTTGGCTGGCGGCCCAGCCGGCGATGGCG
NSP4	ATGAAAAAGATTACCGCTGCTGCTGGTCTGCTGCTCCTCGCTGCCCAGCCGGCGATGGCG
NSP4a	ATGAAAAAGATTCTGCTGCTCCTCGGTACCGCTGCTGCTGCTGCCCAGCCGGCGATGGCG
NSP4b	ATGAAAAAGATTCTGCTGCTCCTCCTGCTGCTCCTCCTGCTCGCCCAGCCGGCGATGGCG
NSP4c	ATGAAAAAGATTGCTGCTGCTGCTGCGGCGGCGGCGGCTGCGGCCCAGCCGGCGATGGCG
NSP5	ATGAAATACCTGCTGCCGTGGCTGGCGCTGGCTGGTTTAGTTTTAGCCCAGCCGGCGATGGCG
NSP6	ATGAAATACCTGCTGCCGACCGCTGCTGCTGGTCTGCTGCTCCTCGCTGCGTTTAGCGCATCGGCG

### Micro 24 fed-batch culture processes

Small volume fed-batch cultures (3 mL working volume) were performed in a Micro 24 bioreactor system (Applikon Biotechnology, Forster City, CA, USA). Feed solutions and other supplements were sterilized by autoclaving or filtration through a 0.22-μm pore size filter, while the rich growth culture medium [20.3 g/L yeast extract (BioSpringer, Milwaukee, WI, USA), 10.1 g/L sodium sulfate anhydrous (JT baker, Center Valley, PA, USA) and 7 g/L K_2_HPO_4_ (JT baker, Center Valley, PA, USA)] was separately autoclaved and added later under aseptic conditions. The culture was initiated by inoculation of 2.8% culture volume into the rich growth medium containing 50 μg/mL of kanamycin (Sigma, St. Louis, MO, USA), 7.6 g/L of trace metal cocktail solution (55 g/L sodium citrate dehydrate (Sigma, St. Louis, MO, USA), 27 g/L FeCl_3_·6H_2_O (Sigma, St. Louis, MO, USA), 0.5 g/L CoCl_2_·6H_2_O (Sigma, St. Louis, MO, USA), 0.5 g/L Na_2_MoO_4_·2H_2_O (Sigma, St. Louis, MO), 0.95 g/L CuSO_4_·5H_2_O (Sigma, St. Louis, MO, USA), 1.6 g/L MnCl_2_·4H_2_O (Sigma, St. Louis, MO, USA), 1.3 g/L ZnCl_2_ (Sigma, St. Louis, MO, USA) and 2 g/L CaCl_2_ (Sigma, St. Louis, MO, USA) and 0.8% culture volume of the glycerol and Epsom salt solution [315 g/L of glycerol (Sigma, St. Louis, MO, USA) and 31.4 g/L of MgSO_4_ (Sigma, St. Louis, MO, USA)]. During the fed-batch cultivation, the impeller speed was initially set to 800 rpm and later controlled to keep the dissolved oxygen level (DO) at 60% saturation. In fed-batch mode, 55% (v/v) glycerol for the carbon source and 33% (w/v) yeast extract were used as feed solutions. Recombinant AT_H35L_ gene expression was induced by addition of 0.5 mM isopropyl-β-D1-thiogalactopyranoside (IPTG) (Biovectra, Charlottetown, PE, USA) when the cell reached at an optical cell density (OD_600_) of 10. Cell culture was continued at 30 °C for an additional 14 h after induction. Cultured cells were collected at 14 h post-induction, and cell culture medium was collected from the harvest samples by centrifugation at 13,300*g* for 5 min for analysis and quantification of extracellular AT_H35L_. Periplasmic and cytoplasmic fractions were prepared from cell lysate from the harvest samples using PeriPreps™Periplasting kit (Epicentre, Madison, WI, USA).

### Fermentor fed-batch culture processes

Large volume fed-batch cultures were performed in a DasGip fermentor (SaniSure Inc, Moorepark, CA, USA) with 1 L working volume. Feed solutions, culture medium and other supplements were prepared as previously described for the small volume fed-batch culture processes. The culture was initiated by inoculation of 2.8% of culture volume into the prepared culture medium containing 50 μg/mL of kanamycin (Sigma, St. Louis, MO, USA), 7.6 g/L of trace metal cocktail solution, 15.8 g/L of glycerol (Sigma, St. Louis, MO, USA) and 1% (v/v) P2000 antifoam (Alfa Aesar, Reston, VA, USA) solution. Air space velocity was 1 vvm and the temperature was maintained at 30 °C. Ammonium hydroxide (23.5% v/v) (Sigma, St. Louis, MO, USA) and glacial acetic acid (50% v/v) solutions (Sigma, St. Louis, MO, USA) were used to maintain cultures at pH 7. During batch experiments, the impeller speed was initially set to 1200 rpm and later controlled to keep the DO at 60% saturation. In fed-batch mode, 55% (v/v) of glycerol for the carbon source and 33% (w/v) of yeast extract solutions were used as feed solutions. During feeding, the impeller speed was maintained constant at 1200 rpm, while the DO saturation was automatically kept at 60%. Recombinant AT_H35L_ gene expression was induced by addition of 0.5 mM IPTG (Biovectra, Charlottetown, PE, USA) at an optical cell density of 80 (OD_600_). After induction, cell culture was continued in fed-batch mode at 30 °C for an additional 12 h. Cultured cells were collected at different time points post-induction to determine the profiles of secreted AT_H35L_ protein, osmolality and the concentration of glycerol and acetate in culture medium.

### Analyses

#### SDS-PAGE

For SDS-PAGE analysis, supernatants from the harvest samples were treated with 4× Bolt^®^ LDS sample buffer (Life Technology, Frederick, MD, USA) which contains both lithium dodecyl sulfate as a denaturing agent and dithiothreitol (DTT) (Life Technology, Frederick, MD, USA) as a reducing agent. All samples were heated at 90 °C for 3 min before loading on SDS-PAGE gels. SDS-PAGE was performed using 10% pre-cast Bis–Tris NuPAGE SDS gels (Life Technology, Frederick, MD, USA). Electrophoresis was performed at a constant 200 V for 45 min in MOPS running buffer under denaturing conditions (Life Technology, Frederick, MD, USA). The separated protein bands were visualized by staining with the Simply Blue Safe Stain solution (Life Technology, Frederick, MD, USA) or transferred onto nitrocellulose membranes for Western blot analysis.

#### Detection of AT_H35L_ protein by Western blot

Harvested samples were separated by SDS-PAGE under denaturing conditions and transferred onto nitrocellulose membranes using an iblot transfer kit (Life Technology, Frederick, MD, USA). Membranes were incubated in blocking buffer (5% skim milk in 0.1% TTBS buffer) at room temperature for 1–1.5 h, then incubated with 1 μg/mL of anti-Staphylococcal alpha hemolysin toxin mAb (LC10 mAb; MedImmune Inc, Gaithersburg, MD, USA) in 0.1% TTBS buffer at room temperature for 1 h or at 4 °C overnight. After washing membranes with 0.1% TTBS at room temperature, the membrane was incubated with 1 μg/mL of HRP conjugated Goat anti Mouse Ab (Bethyl Laboratories, Montgomery, TX, USA) in 0.1% TTBS at room temperature for 1 h. All membranes were then visualized with the SuperSignal West Pico Chemiluminescent Substrate (Pierce, Rockford, IL, USA) and scanned on a ChemiDoc XRS™ system (Biorad, Hercules, CA, USA).

#### Quantification of extracellular AT_H35L_ protein

The concentration of AT_H35L_ in cell culture medium was determined by a customized Octet assay. The assay was performed on the Octet QKe (Fortebio, MenloPark, CA, USA) using anti-Staphylococcal alpha hemolysin toxin mAb (LC10 mAb; MedImmune Inc, Gaithersburg, MD, USA) to capture the AT_H35L_ protein. All samples were diluted at a 1:10 and 1:20 ratio with the kinetics buffer (Fortebio, MenloPark, CA, USA) in a 96-well plate (Corning, Tewksbury, MA, USA). For the Octet assay process, LC10 mAb was bound to the Protein A biosensor (Fortebio, MenloPark, CA, USA) at 300 rpm for 300 s, followed by the base line step in the kinetics buffer at 300 rpm for 60 s, the sample association step at 300 rpm for 150 s, and the dissociation step in the kinetics buffer at 300 rpm for 60 s in the basic kinetic mode. Experimental curves were recorded for the individual samples, and data was processed and analyzed using the Octet data analysis software 7.0 (Fortebio, MenloPark, CA, USA). Finally, samples were quantified by comparison with a standard curve generated from serial dilutions of affinity-purified AT_H35L_ protein. Standard deviation values between the 1:10 and 1:20 diluted samples were less than 10%.

#### Purification of AT_H35L_ from cultivation medium

For N-terminal peptide sequence analysis, extracellular AT_H35L_ in cultivation medium was purified using a combination of ammonium sulfate precipitation and Poros XS cation exchange chromatography (Life Technologies, Grand Island, NY, USA). Briefly, the AT_H35L_ culture was harvested by centrifugation at 850*g* for 30 min to remove the cells. The resulting culture medium was adjusted to pH 5.2 with 1 M acetic acid (Sigma, St. Louis, MO, USA), and centrifuged at 9500*g* for 15 min to remove precipitant. The supernatant was then purified using ammonium sulfate precipitation, followed by using Poros XS cation exchange resin (Life Technologies, Grand Island, NY, USA).

#### N-terminal polypeptide sequencing

Purified AT_H35L_ was fractionated by SDS-PAGE and then transferred onto a nitrocellulose membrane using an iblot transfer kit (Life Technology, Frederick, MD, USA). The N-terminal amino acid sequencing analyses of the isolated AT_H35L_ samples were performed by Covance (Greenfield, IN, USA) using an automated protein/peptide sequencing system.

## Results

### Screening homologous and heterologous signal peptides for enhanced AT_H35L_ secretion in *E. coli*

Since the selection of the signal peptide has a major impact on recombinant protein secretion in *E. coli* systems (Sjostrom et al. 1987), three *E. coli* signal peptides, two from the Sec pathway (pelBss and phoAss) and one from the SRP pathway (dsbAss), were screened to identify a signal peptide for efficient AT_H35L_ secretion. It has been previously shown that some proteins are successfully secreted using their native signal peptides in heterologous expression systems (Rigi et al. 2014; Shahhoseini et al. 2003). Therefore, we also assessed the native signal peptide of AT (NTss) even though the structural elements of signal peptides from extracellular proteins are different between *S. aureus* and *E. coli.* A summary of the signal peptides screened is shown in Table 2. Recombinant cell strains containing different signal peptide-AT_H35L_ fusion constructs were cultured in fed-batch conditions using a Micro24 system. The relative secretion levels of AT_H35L_ protein into the cell culture medium were compared by Western blot analysis using purified mature AT_H35L_ as a reference. The molecular weight of AT_H35L_ precursor protein is approximately 3 kDa higher than mature AT_H35L_ due to the presence of the signal peptide. Precursor and mature AT could therefore be distinguished by their size difference on SDS-PAGE gels.

Analysis of cell culture medium, cytoplasmic and periplasmic samples revealed that mature AT_H35L_ protein was secreted into the culture medium as well as into the periplasmic space after export from the cytoplasm. Although some of soluble AT_H35L_ still remained in the cytoplasm, more than 50% of expressed AT_H35L_ protein for the *E. coli* signal peptides was secreted into the periplasmic space and the culture medium (Additional file 1: Figure S3).

**Fig. 1:**
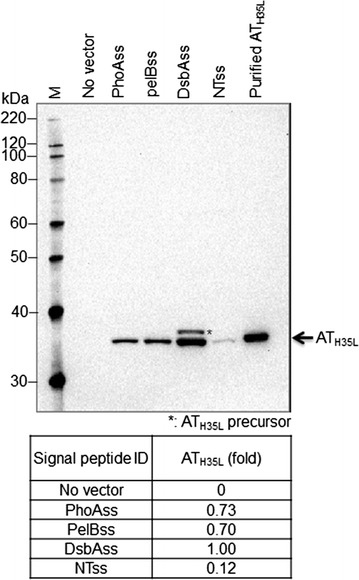
Western blot analysis of cell culture medium from AT_H35L_ signal sequence variants in a Micro24 fed-batch process. *Arrow* indicates released mature AT_H35L_ protein from live cells and *asterisk* indicates leaked AT_H35L_ precursor from dead cells. Purified AT_H35L_ protein was used as a reference. In the table, the levels of secreted AT_H35L_ protein in culture medium were quantified by the band intensity of AT_H35L_ from SDS-PAGE gel using the Image J 1.50i (Schneider et al. 2012)

Figure 1 shows the relative levels of AT_H35L_ secreted into the culture medium from the different signal peptides. DsbAss showed the highest level of secreted AT_H35L_ product, whereas NTss produced the lowest level. The n-region of NTss has a similar net positive charge (+1.1) to the selected *E. coli* signal peptides pelBss (+1), phoAss (+1) and dsbAss (+2). Furthermore, the length of all four signal peptides was similar. However, NTss has the longer h-region (Table 2) with a low similarity of amino acid sequences with the *E. coli* signal peptides (data not shown). Also, hydrophobicity in the h-domain of NTss is lower (50%) than the *E. coli* signal peptides (>80%). This observation implies that hydrophobicity strongly influences the secretion efficiency of recombinant proteins in *E. coli*. For the *E. coli* signal peptides screened, pelBss and phoAss showed a lower level of secreted AT_H35L_ compared with dsbAss (Fig. 1). The amino acid sequences of these three signal peptides show a high level of similarity using the PRALINE multiple sequence alignment tool (Simossis et al. 2005). In particular, the identity of amino acid sequence between dsbAss and pelBss was as high as 45% (data not shown). Therefore, to investigate the impact on the AT_H35L_ secretion of individual signal peptide domains and to improve the secretion efficiency of AT_H35L_, novel signal peptides were designed by modifying the native signal peptide sequences.

### Design and screening of novel signal peptides for improving AT_H35L_ secretion

**Fig. 2:**
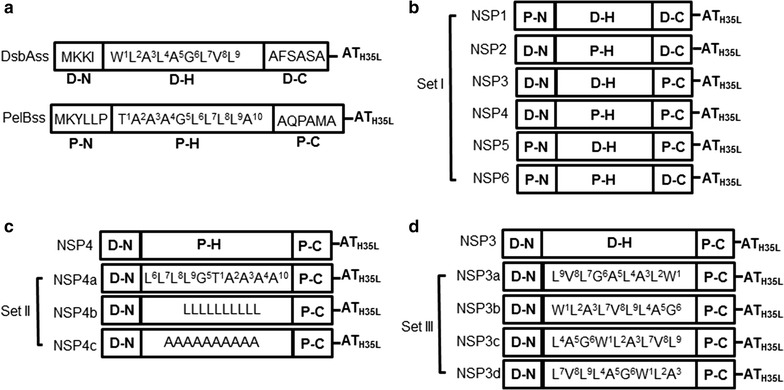
Schematic representation of the novel signal peptide constructions used to express secreted AT_H35L_. **a** DsbAss and pelBss used as parental signal peptides. D–N, D–H and D–C represent the n-, h- and c-regions of dsbAss, respectively and P–N, P–H and P–C represent the n-, h- and c-regions of pelBss, respectively. **b** Set I novel signal peptides, NSP1–NSP6, were created by shuffling the n-, h- and c-regions from dsbAss and pelBss. **c** Set II novel signal peptides. NSP4a–NSP4c were created by modifying the h-region of NSP4 by changing the position of amino acid residues or substituting residues with polyleucine or polyalanine. **d** Set III novel signal peptides. NSP3a–NSP3d were created by modifying the position of amino acid residues in the h-region of NSP3. In **c**, **d** *Numbers* next to individual amino acids in the h-region indicate the original position in the h-region in (**a**)

With regard to Set I novel signal peptides, dsbAss and pelBss were selected to initiate the design of new signal peptides. In spite of the high level of amino acid sequence identity between the dsbAss and pelBss, these signal peptides showed different AT_H35L_ secretion efficiencies. Six novel signal peptides, NSP1-6, were created by rearranging individual domains of dsbAss and pelBss and cloned in-frame into the AT_H35L_ expression vector (Table 2; Fig. 2b). After transformation of recombinant plasmids into BL21 Star™ (DE3), recombinant cells were cultured in 1L scale fed-batch conditions to evaluate the secretion of AT_H35L_.

**Fig. 3:**
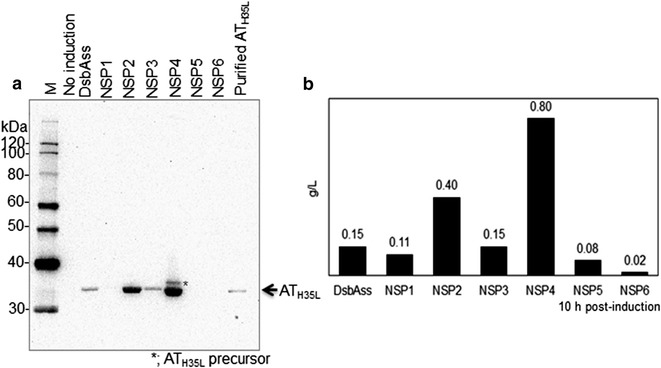
Western blot and titer analyses of secreted AT_H35L_ by Set I novel signal peptides in cell culture medium in 1L fed-batch bioreactor. **a** Western blot analysis of the secreted AT_H35L_ protein. *Arrow* indicates secreted AT_H35L_ protein and *asterisk* indicates leaked AT_H35L_ precursor from dead cells. **b** Quantification of AT_H35L_ protein in cell culture medium at 10 h post-induction

The data in Fig. 3 show that NSP2 and NSP4 increased the secretion of AT_H35L_ into cell culture medium by 2.5-fold (0.4 g/L) and fivefold (0.8 g/L) respectively compared with dsbAss (0.15 g/L) at 10 h post-induction. Intriguingly, these two novel signal peptides share the same n- and h-domains in their structures (D–N and P–H), but not the c-domains (Table 2; Fig. 2b). The observed improvement in secretion suggests that combination of the n-domain of dsbAss (D–N) and the h-domain of pelBss (P–H) results in a favorable structure for efficient translocation of AT_H35L_ across the cytoplasmic membrane. Although both signal peptides improved the AT_H35L_ secretion into cell culture medium, the impact on secretion efficiency of AT_H35L_ was different depending on the c-domain in their structure. The AT_H35L_ secretion was more efficient when the c-domain of pelBss (P–C) was combined with D–N and P–H domains in NSP4 (Figs. 2b, 3).

In contrast to NSP2 and NSP4, the level of secreted AT_H35L_ in cell culture medium was reduced for NSP6 (0.02 g/L) compared with dsbAss (0.15 g/L), even though NSP6 also contains the h-domain of pelBss like NSP2 and NSP4 (P–H; Fig. 2b) and the same c-domain as NSP2 (D–C; Fig. 2b). Interestingly, NSP1 and NSP5 contain the n-domain of pelBss like NSP6 (P–N; Fig. 2b) and the secreted AT_H35L_ in cell culture medium was also reduced for these two signal peptides.

Unlike NSP1, NSP5 and NSP6, AT_H35L_ secretion was not reduced for NSP2, NSP3 and NSP4 in comparison to dsbAss. NSP2, NSP3 and NSP4 all contain the n-domain of dsbAss in their structure (D–N in Fig. 2b). NSP3 shares the same n-and c-domains (D–N and P–C) with NSP4, but contains a different h-domain than NSP2 and NSP4 (D–H for NSP3 and P–H for NSP2 and NSP4). Figure 3 demonstrates that NSP3 did not improve the yield of secretory AT_H35L_ (0.15 g/L), whereas NSP4 had the highest yield of secretory AT_H35L_ (0.8 g/L). The present data denote that the n-domain of dsbAss (D–N) is a favorable for translocating AT_H35L_ across the cytoplasmic membrane. Moreover, AT_H35L_ secretion is more efficient when D–N and P–H domains are combined (NSP2 and NSP4 in Fig. 3) compared with the combination of D–N and D–H (NSP3 in Fig. 3). In addition, we detected AT_H35L_ precursor protein by Western blot only from NSP4, although NSP3, NSP4 and NSP5 all contain the same c-domain (P–C) which determines signal peptide cleavage. One explanation is that that the detected AT_H35L_ precursor protein was released from dead cells.

In Fig. 3, NSP1, NSP5 and NSP6 have the same n-domain (P–N), but not the h-domain. The secretion of AT_H35L_ for NSP1 is less reduced than NSP5 and NSP6 although NSP1 has the same n- and h-domains with NSP5 (P–N and D–H). In Fig. 2b, NSP1 is composed of homologous h- and c-domains (D–H and D–C) while NSP5 and NSP6 are composed of heterologous h- and c-domains (D–H and P–C or P–H and D–C). We hypothesized that the signal peptide structure containing homologous h-and c-domains is more efficient for secretion of recombinant proteins. To verify this hypothesis, we also compared the AT_H35L_ secretion between NSP2 and NSP4, and the result shows that these two signal peptide also have similar structural features for the secretion of AT_H35L_ (P–H and D–C for NSP2 and P–H and P–C for NSP4). Further studies are still required to determine if the amino acid residues in the h- and c-domains influence each other to affect AT_H35L_ secretion. However, in comparison among signal peptides containing the same n-domain, the data clearly showed that the composition of homologous h- and c-domains was more efficient for AT_H35L_ secretion than the composition of heterologous h- and c-domains (NSP1>NSP5 or NSP6 and NSP4>NSP2 or NSP3 in Fig. 3).

### Effects of altering the position of amino acids in the h-domain on the secretion of AT_H35L_

Two further series of novel signal peptides were created by modifying the position of amino acids in the h-regions of NSP3 and NSP4 in order to investigate whether modification of the hydrophobic amino acid position in the h-region affected the secretion efficiency of the AT_H35L_ protein. NSP3 and NSP4 mutants were generated by shuffling the position of amino acids in the h-region or substituting all the h-region amino acids in the case of NSP4b and NSP4c (Fig. 2a, c). Cell culture medium samples from NSP3 (Set III) and NSP4 (Set II) mutant constructs were collected at 12 h post-induction for evaluating the productivity of secreted AT_H35L_.

**Fig. 4:**
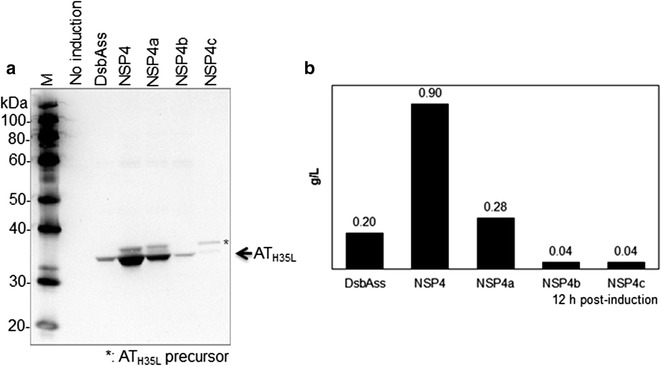
Screening of Set II novel signal peptides for AT_H35L_ secretion in 1L fed-batch bioreactor. **a** Western blot analyses of secreted AT_H35L_ protein in cell culture medium. **b** Quantification of extracellular AT_H35L_ protein in cell culture medium at 12 h post-induction

For the Set II signal peptides (Fig. 2c; Table 2), NSP4a was created by rearranging the position of hydrophobic residues in the h-domain in order to investigate whether the position of hydrophobic residues affects secretion of AT_H35L_. Since total hydrophobicity of the h-domain is an important factor in determining the secretion efficiency of a recombinant protein (Hikita and Mizushima 1992a, b; Chou and Kendall 1990), we additionally generated a couple of mutants, NSP4b and NSP4c. Total hydrophobicity of these mutants were changed by replacing residues of the h-domain with polyleucine or polyalanine (Fig. 2c). The result shown in Fig. 4 indicates that NSP4 mutant signal polypeptides reduced overall AT_H35L_ secretion levels. NSP4 produced 0.9 g/L of secretory AT_H35L_ in cell culture medium, but NSP4a reduced the yield of secretory AT_H35L_ to 0.28 g/L. Also, the yield of secretory AT_H35L_ for both NSP4b and NSP4c was significantly reduced to 0.04 g/L regardless of increasing or diminishing total hydrophobicity of the h-domain (Fig. 4b). In Fig. 4a, mainly unprocessed AT_H35L_ precursor was detected by Western blot for NSP4c. The total hydrophobicity of the h-domain in NSP4c was significantly decreased and it seems that NSP4c_AT_H35L_ was not translocated successfully across the inner membrane from the cytoplasmic space. Most likely, the accumulated AT_H35L_ precursors in the cytoplasmic space leaked out from the intracellular space after cell death.

**Fig. 5:**
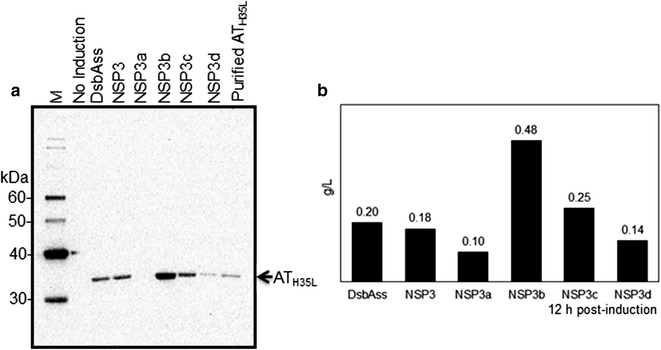
Screening of Set III novel signal peptides for AT_H35L_ secretion in 1L fed-batch bioreactor. **a** Western blot and **b** quantification of extracellular AT_H35L_ protein in cell culture medium at 12 h post-induction

For NSP3, there was no change of the AT_H35L_ secretion efficiency compared to dsbAss despite having the same n- and c-domains of NSP4 in its structure (Figs. 2b, 3). In the study of the Set II NSP4 mutants, the secretion efficiency of AT_H35L_ was significantly affected by the position of hydrophobic residues in the h-region (NSP4a in Figs. 2c, 4) and NSP4 did not affect the level of translation of this protein (Additional file 1: Figure S3). Thus, four mutants of NSP3, Set III, were generated by rearranging the location of amino acid residues in the h-domain in order to investigate the relationship between the amino acid position and the secretion efficiency of AT_H35L_. Amino acid residues in the h-domain was grouped and each group contained three amino acid residues. The groups were shuffled to relocate their position (Fig. 2a, d). The original position order of each amino acid in the h-region is numbered next to amino acids in the Table 2 and Fig. 2d. The secretion of AT_H35L_ by the Set III signal peptides is shown in Fig. 5. NSP3b and NSP3c improved the yield of secretory AT_H35L_ by 2.7-fold (0.48 g/L) and 1.4-fold (0.25 g/L) respectively compared with NSP3 (0.18 g/L). In contrast, NSP3a and NSP3d reduced the yield of secretory AT_H35L_ to 0.1 g/L and 0.14 g/L, respectively (Fig. 5b). In Western blot analysis of Set III signal peptides, AT_H35L_ precursor was not detected from any of NSP3 mutants (Fig. 5a).

### Determining the optimal induction period for NSP4_AT_H35L_ secretion

Figure 3 shows that the secretion of AT_H35L_ into cell culture medium by NSP4 is approximately fivefold higher than dsbAss at 10 h post-induction. At the same time, AT_H35L_ precursor protein was additionally detected from NSP4_AT_H35L_ by Western blot, whereas there was no detection of AT_H35L_ precursor from the other signal peptides. Thus, we performed a series of fed-batch cell culture experiments to determine the optimal induction time for maximizing the secretion of AT_H35L_ while minimizing the contamination with AT_H35L_ precursor.

**Fig. 6:**
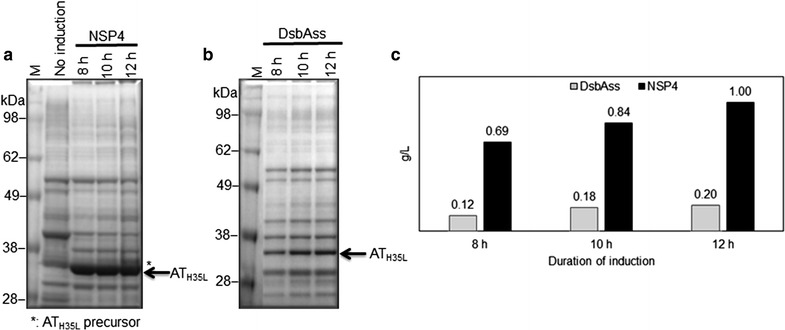
Optimization of induction time for extracellular AT_H35L_ production. Cell culture medium samples were collected every 2 h from 8 h post-induction. SDS-PAGE analysis of extracellular AT_H35L_ production by NSP4 (**a**) and by dsbAss (**b**); **c** Comparison of extracellular AT_H35L_ production between dsbAss and NSP4 at various induction times

Figure 6 shows the secretion of AT_H35L_ from NSP4 and dsbAss into cell culture medium at various induction time points. Secretion of NSP4_AT_H35L_ increased from 0.69 g/L at 8 h post-induction up to 1 g/L at 12 h post-induction. In comparison with NSP4, the productivity of secreted AT_H35L_ for dsbAss increased from 0.12 g/L at 8 h post-induction up to 0.2 g/L at 12 h post-induction. The yield of secreted AT_H35L_ for NSP4 was 5.7-fold higher than with dsbAss at 8 h post-induction and was consistently increased by approximate fivefold at 12 h post-induction compared with dsbAss. For NSP4_AT_H35L_, the AT_H35L_ precursor was not detected at 8 h post-induction, but started to appear after 10 h post-induction. In the cell growth profile, the cell growth rate of NSP4_AT_H35L_ was gradually reduced from 8 h post-induction (Additional file 1: Figure S2). In contrast to NSP4, the cell growth rate and the productivity of secreted AT_H35L_ for dsbAss_AT_H35L_ were consistently increased up to 12 h post-induction (Figs. 6b and Additional file 1: Figure S2). After 12 h post-induction, the productivity of secreted AT_H35L_ for dsbAss did not increase (data not shown). Additionally, we did not detect AT_H35L_ precursor in the cell culture medium of dsbAss_AT_H35L_ up to 12 h post-induction (Fig. 6a). Thus, the optimal induction times for secretory AT_H35L_ from NSP4_AT_H35L_ and dsbAss_AT_H35L_ were at 8 and 12 h post-induction respectively when the yield of secretory AT_H35L_ for NSP4 was ≥3.5-fold higher than for dsbAss. Therefore, the present data show that NSP4 substantially improved the levels of AT_H35L_ secretion in a shorter induction time compared with dsbAss.

In the secretion of recombinant proteins, the site and consistency of cleavage of the signal peptide by the signal peptidase is an important product quality attribute. To verify the cleavage of AT_H35L_ from NSP4, secreted AT_H35L_ in culture medium was purified and analyzed by N-terminal peptide sequencing (Additional file 1: Figure S1). The data confirmed that the novel signal peptide, NSP4, was correctly cleaved from AT_H35L_ precursor protein and mature AT_H35L_ protein in cell culture medium was released from the periplasmic space (Additional file 1: Table S1).

## Discussion

The goals of our study were to evaluate the impact of different signal sequences on the secretion of AT_H35L_ into cell culture medium and to increase the secretion of properly processed AT_H35L_ into cell culture medium. In our study, a comparative analysis by Western blot showed that dsbAss was the most effective in directing the secretion of AT_H35L_ into cell culture medium among the initial four signal peptides tested. In contrast, pelBss showed the lowest secretion efficiency of AT_H35L_ among *E. coli* signal peptides (Fig. 1), although amino acid sequences of these two signal peptides are 45% identical using Needleman–Wunsch algorithm (Needleman and Wunsch 1970). This result suggests that different domain compositions of signal peptides can lead to different secretion efficiencies of recombinant proteins despite the high degree of similarity between different signal peptide sequences.

Since the individual domains of the signal peptide have different roles in protein targeting and translocation (Choi and Lee 2004; Lehnhardt et al. 2012), we hypothesized that the secretion efficiency of a recombinant protein can be influenced by modifying the signal peptide domains. In screening the Set I novel signal peptides to investigate this hypothesis, NSP2, NSP3 and NSP4 improved or maintained the secretion of AT_H35L_ into cell culture medium (Fig. 3). In contrast, NSP1, NSP5 and NSP6 reduced the AT_H35L_ secretion (Fig. 3). Several studies have shown that the net positive charge of basic residues in the n-domain is an important feature the highly basic n-domain promotes the interactions of the n-domain with SRP, influencing the translocation of a recombinant protein and removing basic residues from signal peptides reduced the rate of recombinant protein export (Hikita and Mizushima 1992a, b; Low et al. 2013; Nesmeyanova et al. 1997; Nilsson et al. 2015; Peterson et al. 2003; Tian and Bernstein 2009). Consistent with these observations, in this study, the n-domain of dsbAss has higher positive net charge (+2) than the n-domain of pelBss (+1), and signal peptides containing the n-domain of dsbAss (NSP2, NSP3 and NSP4) showed more favorable translocation of AT_H35L_.

NSP2, NSP3 and NSP4 contain a favorable n-domain (D–N) for AT_H35L_ secretion (Figs. 2b and 3). Nevertheless, these signal peptides varied in the secretion of AT_H35L_ and only NSP2 and NSP4 positively impacted the AT_H35L_ secretion over dsbAss (Fig. 3). One of their structural differences is that NSP2 and NSP4 have the h-domain of pelBss (P–H) and NSP3 has the h-domain of dsbAss (D–H). Previous studies have shown that the h-domain also plays a critical role in its secretion activity and total hydrophobicity of the h-region is a key determinant of secretion efficiency (Choi and Lee 2004; Duffy et al. 2010; Hikita and Mizushima 1992a; Sjostrom et al. 1987; Zanen et al. 2005). Additionally, increased total hydrophobicity in the h-domain of signal peptides has been demonstrated to improve recombinant protein secretion (Chou and Kendall 1990; Jonet et al. 2012; Klatt and Konthur 2012). Thus, we analyzed total hydrophobicity values of the h-domain for the selected *E. coli* signal peptides including NSP4b and NSP4c (Table 4). Interestingly, the h-domain of NSP2 and NSP4 (P–H) has a lower total hydrophobicity than the h-domain of NSP3 (D–H). Since NSP2, NSP3 and NSP4 share the same n-domain, variation in the basic amino acid residues in the n-domain cannot explain the differences in AT_H35L_ secretion observed with these signal peptides. Similarly, the h-domain of phoAss has the highest hydrophobicity among the selected *E. coli* signal peptides (phoAss > dsbAss > pelBss; Table 4), but dsbAss showed better AT_H35L_ secretion efficiency than phoAss (Fig. 1). Moreover, the AT_H35L_ secretion efficiency was reduced for NSP4b regardless of the increased total hydrophobicity by using substitution of amino acid residues in the h-domain with polyleucine (Fig. 2c; Table 4). This observed lack of correlation of overall h-domain hydrophobicity with secretion may be associated with the composition of the hydrophobic amino acid residues. Several studies have noted that the composition of hydrophobic amino acids in the h-domain are not random and the organization of hydrophobic residues in the h-domain of signal peptide is important for leading the translocation of a recombinant protein in secretion process (Duffy et al. 2010; Lehnhardt et al. 2012). Thus, this would explain why NSP4b with its homogeneous hydrophobic h-domain, significantly decreased the secretion of AT_H35L_ (Fig. 4; Table 4). Not surprisingly, hydrophobic amino acids in the h-domain are required for secretion because when the NSP4 h-domain hydrophobicity was demolished by replacing all residues in the h-domain with polyalanine in NSP4c, there was failure of AT_H35L_ secretion. This suggests that placing strong hydrophobic amino acid residues in the signal peptide are required for the secretion of a recombinant protein, but the composition of amino acids in the h-domain is more critical than merely increasing total hydrophobicity to improve secretion of recombinant proteins.

**Table 4:** Total hydrophobicity values of the h-domain

Signal peptide ID	kdHydrophobicity^a^	wwHydrophobicity^b^
DsbAss	21.7	3.67
PelBss	21.3	1.41
PhoAss	25.5	2.32
NSP4b	38	5.6
NSP4c	18	−1.7

The total hydrophobicity of the h-domain amino acid sequences were calculated according to ^a^Kyte and Doolittle (1982) and ^b^Wimley and White (1996)

We changed the pattern of amino acid residues in the h-domain whilst maintaining the overall composition and hydrophobicity of the h-domain to investigate whether AT_H35L_ secretion could be improved. In NSP4a, altering the position of individual amino acids significantly impacted the secretion efficiency of AT_H35L_ (Fig. 4). Similarly, NSP3a,b,c and d showed differing secretion efficiencies after rearranging the position of amino acid residues in the h-domain of NSP3 (Fig. 5). In the NSP3b and c mutants, the location of strong hydrophobic residues, such as leucine and valine, at the center of the h-domain or close to the c-domain increased the AT_H35L_ secretion. In contrast, when these hydrophobic residues were located close to the n-domain as in NSP3a and NSP3d, AT_H35L_ secretion was decreased. Consistent with our hypothesis, NSP4a also showed reduced AT_H35L_ secretion when strongly hydrophobic amino acid residues in the h-domain were located close to the n-polar region. This also suggests that hydrophobic residues in the h-domain interact with amino acids in the n- and c-domains.

In conclusion, in designing and testing novel signal peptides we have both improved the secretion of recombinant AT_H35L_ from *E. coli* (Additional file 1: Figure S3) and also increased the understanding of the influence of the composition and interaction of the signal peptide domains on secretion. The data presented here demonstrated that (1) the combination of the n-domain of dsbAss and the h-domain of pelBss is favorable for AT_H35L_ secretion, (2) hydrophobicity in the h-region is a critical component for translocation of recombinant proteins across the cytoplasmic membrane, (3) the composition and arrangement of hydrophobic amino acids in the h-domain influence the secretion efficiency of recombinant proteins. From these studies, we have identified a novel signal peptide (NSP4) that significantly improved AT_H35L_ secretion and decreased the induction time, which are both very beneficial for commercial production although the exact mechanism of the secretion pathway for NSP4_AT_H35L_ is not yet clear. Furthermore, we believe that the novel signal peptides designed in the present study could be used to improve the secretion efficiency of other recombinant proteins in the *E. coli* expression platform.

## Additional file

**Additional file 1: Table S1.** Identified peptide sequences of secreted ATH35L in culture medium. **Figure S1.** SDS-PAGE analysis of purified ATH35L protein. Secreted ATH35L by NSP4 in culture medium was purified and used for N-terminal peptide sequencing analysis. **Figure S2.** The cell growth profiles of NSP4_ATH35L and dsbAss_ATH35L. Recombinant ATH35L gene expression was induced by addition of 0.5 mM IPTG at an optical cell density of 80 (OD600). After induction, cell culture was continued in fed-batch mode at 30 °C for an additional 12 h. Cultured cells were collected at different time points postinduction to determine the profiles of secreted ATH35L protein in culture medium. **Figure S3.** Western blot analysis of cell lysates and cell culture medium from dsbA_ATH35L and NSP4_ATH35L in a Micro24 fed-batch process. (a) Arrow indicates ATH35L protein in cell crude lysates. (b) Arrow indicates released mature ATH35L protein in cell culture medium. Purified ATH35L protein was used as a reference.

## Abbreviations

°C: degree centigrade
AT: alpha hemolysin toxin
AT_H35L_: alpha hemolysin toxin_H35L_ mutant
DsbAss: a bacterial disulfide bond formation protein A signal sequence
DTT: dithiothreitol
*E. coli*: *Escherichia coli*
HRP: horseradish peroxidase
IPTG: isopropyl-β-D1-thiogalactopyranoside
NTss: native signal sequence
NSP: novel signal peptide
PelBss: pectate lyase B signal sequence of Erwinia carotovora CE
PhoAss: alkaline phosphatase signal sequence of *E.coli*
*S. aureus*: *Staphylococcus aureus*
SDS-PAGE: sodium dodecyl sulfate polyacrylamide gel electrophoresis
The c-region: a protease recognition sequence
The h-region: a hydrophobic region
The n-region: an amino-terminal region with a net positive charge
SEC pathway: the Sec-dependent secretion pathway
SRP: the signal peptide recognition particle
TAT pathway: the twin-arginine translocation secretion pathway
TTBS: tris-buffered saline (TBS) and Polysorbate 20
