# Supplementary material for: Novel signal peptides improve the secretion of recombinant Staphylococcus aureus Alpha toxinH35L in Escherichia coli
Source: AMB Express. 2017 May 12;7:93. doi: 10.1186/s13568-017-0394-1 (PMC5427057; doi:10.1186/s13568-017-0394-1)
Supplement: Supplementary file 1 — Additional file 1: Table S1. Identified peptide sequences of secreted ATH35L in culture medium. Figure S1. SDS-PAGE analysis of purified ATH35L protein. Secreted ATH35L by NSP4 in culture medium was purified and used for N-terminal peptide sequencing analysis. Figure S2. The cell growth profiles of NSP4_ATH35L and dsbAss_ATH35L. Recombinant ATH35L gene expression was induced by addition of 0.5 mM IPTG at an optical cell density of 80 (OD600). After induction, cell culture was continued in fed-batch mode at 30 °C for an additional 12 h. Cultured cells were collected at different time points postinduction to determine the profiles of secreted ATH35L protein in culture medium. Figure S3. Western blot analysis of cell lysates and cell culture medium from dsbA_ATH35L and NSP4_ATH35L in a Micro24 fed-batch process. (a) Arrow indicates ATH35L protein in cell crude lysates. (b) Arrow indicates released mature ATH35L protein in cell culture medium. Purified ATH35L protein was used as a reference. [file 13568_2017_394_MOESM1_ESM.pdf]

AMB Express

Supplementary Material

Novel signal peptides improve the secretion of recombinant *Staphylococcus aureus* Alpha toxin$_{H35L}$ in *Escherichia coli*.

SooJin Han[1*] • Shushil Machhi[1] • Mark Berge[1] • Guoling Xi[2] • Thomas Linke[2] • Ronald Schoner[1, 3]

1. Cell Culture and Fermentation Sciences, MedImmune Inc., Gaithersburg, MD 20878, USA
2. Purification Process Sciences, MedImmune Inc., Gaithersburg, MD 20878, USA
3. Current address: BioProcess Sciences, Qilu Puget Sound Biologics, Bothell, WA 98021, USA

Contributor contact information:

*: To whom correspondence should be addressed, at the above address. E-mail: HanS@medimmune.com; Phone: +1-301-398-1857

Shushil Machhi: MachhiS@medimmune.com ; Phone: +1-301-398-2512

Mark Berge: BergeM@medimmune.com; Phone: +1-301-398-6767

Guoling Xi: XiG@medimmune.com; Phone: +1-301-398-4889

Thomas Linke: LinkeT@medimmune.com; Phone: +1-301-398-5606

Ronald Schoner: ron@soundbiologics.com; Phone: +1-317-496-2036

Table S1. Identified peptide sequences of secreted AT_{H35L} in culture medium

The position of amino acid	Released AT_{H35L} from NSP4	AT_{H35L}
1	A	A
2	D	D
3	S	S
4	D	D
5	I	I
6	N	N
7	I	I
8	K	K
9	T	T

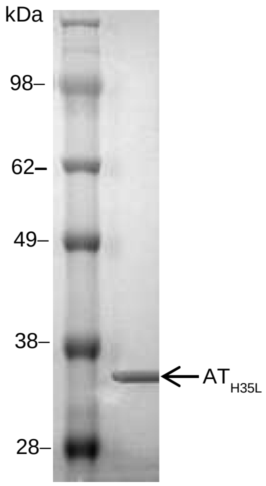

Fig S1. SDS-PAGE analysis of purified AT_{H35L} protein. Secreted AT_{H35L} by NSP4 in culture medium was purified and used for N-terminal peptide sequencing analysis.

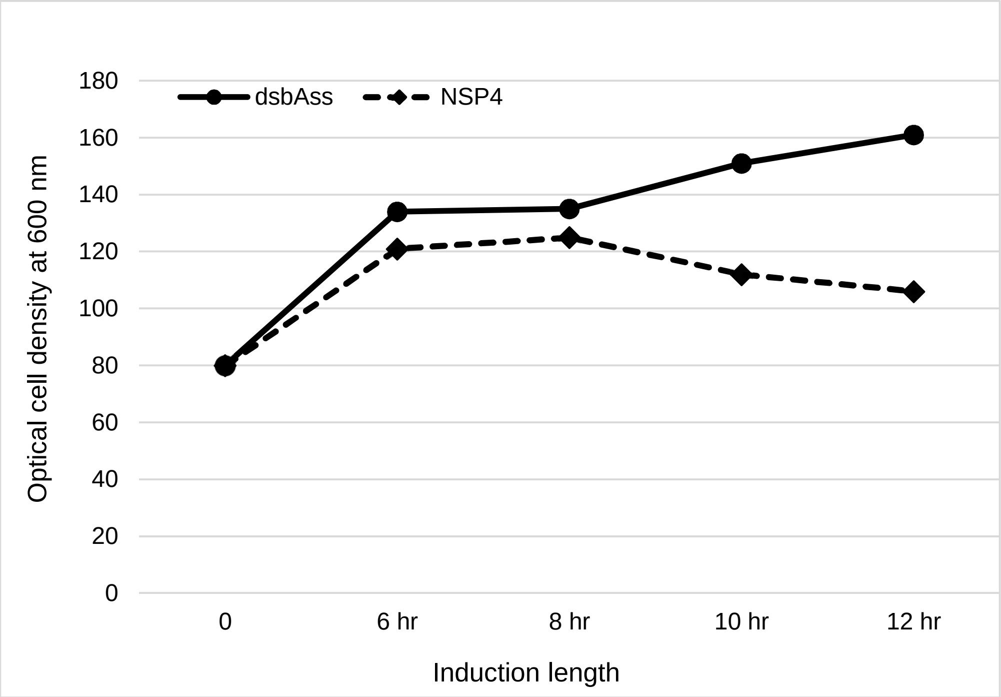

Fig. S2 The cell growth profiles of NSP4_AT_{H35L} and dsbAss_AT_{H35L}. Recombinant AT_{H35L} gene expression was induced by addition of 0.5 mM IPTG at an optical cell density of 80 (OD_{600}). After induction, cell culture was continued in fed-batch mode at 30 °C for an additional 12 h. Cultured cells were collected at different time points post-induction to determine the profiles of secreted AT_{H35L} protein in culture medium.

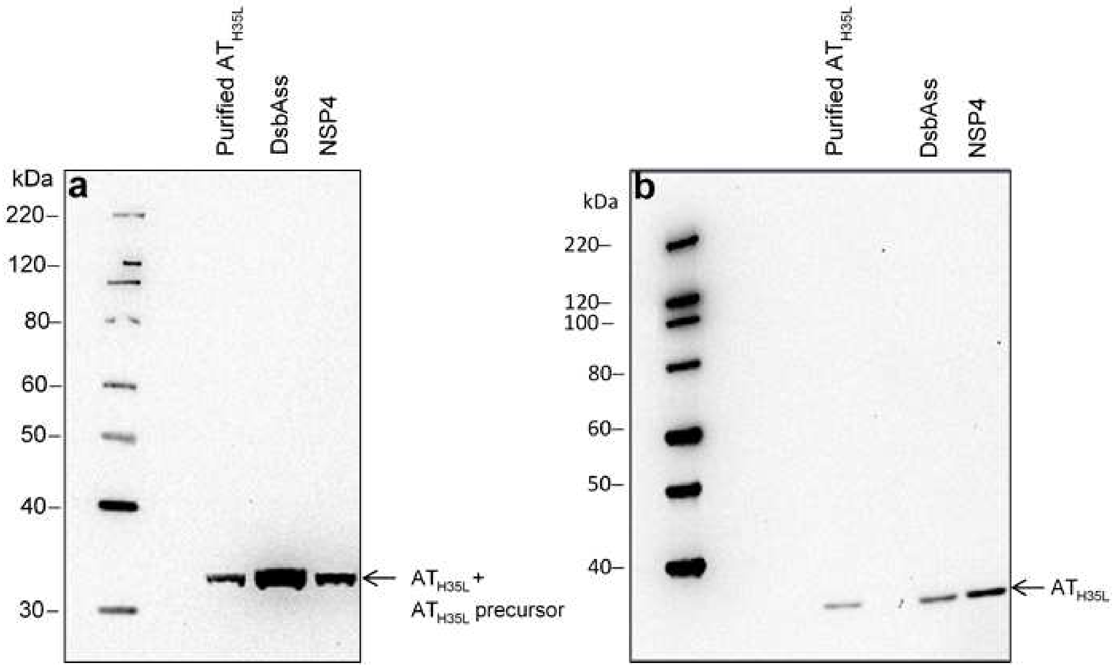

Fig S3. Western blot analysis of cell lysates and cell culture medium from dsbA_AT_{H35L} and NSP4_AT_{H35L} in a Micro24 fed-batch process. (a) Arrow indicates AT_{H35L} protein in cell crude lysates. (b) Arrow indicates released mature AT_{H35L} protein in cell culture medium. Purified AT_{H35L} protein was used as a reference.
